# Withaferin A alleviates fulminant hepatitis by targeting macrophage and NLRP3

**DOI:** 10.1038/s41419-020-03243-w

**Published:** 2021-02-11

**Authors:** Yangliu Xia, Ping Wang, Nana Yan, Frank J. Gonzalez, Tingting Yan

**Affiliations:** 1 Laboratory of Metabolism, Center for Cancer Research, National Cancer Institute, National Institutes of Health, Bethesda, MD 20892 USA; 2 School of Life and Pharmaceutical Sciences, Dalian University of Technology, Panjin, 124221 China; 3 Institute of Interdisciplinary Integrative Medicine Research, Shanghai University of Traditional Chinese Medicine, Shanghai, 201203 China; 4 State Key Laboratory of Natural Medicines, Key Laboratory of Drug Metabolism and Pharmacokinetics, China Pharmaceutical University, Nanjing, 210009 Jiangsu China

**Keywords:** Fatty acids, Metabolic disorders

## Abstract

Fulminant hepatitis (FH) is an incurable clinical syndrome where novel therapeutics are warranted. Withaferin A (WA), isolated from herb *Withania Somnifera*, is a hepatoprotective agent. Whether and how WA improves D-galactosamine (GalN)/lipopolysaccharide (LPS)-induced FH is unknown. This study was to evaluate the hepatoprotective role and mechanism of WA in GalN/LPS-induced FH. To determine the preventive and therapeutic effects of WA, wild-type mice were dosed with WA 0.5 h before or 2 h after GalN treatment, followed by LPS 30 min later, and then killed 6 h after LPS treatment. To explore the mechanism of the protective effect, the macrophage scavenger clodronate, autophagy inhibitor 3-methyladenine, or gene knockout mouse lines NLR family pyrin domain containing 3 (*Nlrp3)*-null, nuclear factor-erythroid 2-related factor 2 (*Nrf2*)-null, liver-specific AMP-activated protein kinase (*Ampk)a1* knockout (*Ampka1*^ΔHep^) and liver-specific inhibitor of KB kinase β (*Ikkb*) knockout (*Ikkb*^ΔHep^) mice were subjected to GalN/LPS-induced FH. In wild-type mice, WA potently prevented GalN/LPS-induced FH and inhibited hepatic NLRP3 inflammasome activation, and upregulated NRF2 and autophagy signaling. Studies with *Nrf2*-null, *Ampka1*^ΔHep^, and *Ikkb*^ΔHep^ mice demonstrated that the hepatoprotective effect was independent of NRF2, hepatic AMPKα1, and I*κκ*B. Similarly, 3-methyladenine cotreatment failed to abolish the hepatoprotective effect of WA. The hepatoprotective effect of WA against GalN/LPS-induced FH was abolished after macrophage depletion, and partially reduced in *Nlrp3*-null mice. Consistently, WA alleviated LPS-induced inflammation partially dependent on the presence of NLRP3 in primary macrophage in vitro. Notably, WA potently and therapeutically attenuated GalN/LPS-induced hepatotoxicity. In conclusion, WA improves GalN/LPS-induced hepatotoxicity by targeting macrophage partially dependent on NLRP3 antagonism, while largely independent of NRF2 signaling, autophagy induction, and hepatic AMPKα1 and I*κκ*B. These results support the concept of treating FH by pharmacologically targeting macrophage and suggest that WA has the potential to be repurposed for clinically treating FH as an immunoregulator.

## Introduction

Fulminant hepatitis (FH) is a global life-threatening clinical syndrome with no curable drugs and measures available other than liver transplantation, and thus novel drug discovery is imperative^1,2^. In the clinic, lipopolysaccharide (LPS) released from intestinal bacterial stimulates macrophage to release tumor necrosis factor (TNF)-α^3–5^. While LPS alone only induces systematic inflammation and LPS pretreatment can even ameliorate hepatotoxicity^3,6,7^, LPS-induced hepatotoxicity is usually enhanced when livers are pre-exposed to various other hepatic insults that are frequently involved with humans in the clinic, such as viruses, toxins and alcohol^1,2,8^. These hepatic insults could deplete hepatic glutathione levels^9,10^ and/or inhibit the protein and RNA transcription, both of which could sensitize hepatocytes to TNF-α-induced apoptosis^11–13^. To mimic the clinical FH, the D-galactosamine (GalN) and LPS-induced acute liver injury model has been established in mice^7,14,15^. GalN/LPS induces FH by GalN-induced inhibition of protein and RNA synthesis that sensitizes hepatocytes to TNF-α-induced apoptosis^16^, followed by TNF-α release through LPS-stimulated macrophage^14^. Therefore, FH progresses through two processes, sensitizing livers by GalN, and LPS activation of macrophage to release TNF-α, which together contribute to extensive TNF-α-induced hepatocyte apoptosis^17,18^.

Many targets in the liver and macrophage, which could contribute to hepatocellular stress including oxidative stress, inflammation and hepatocyte death, mediate GalN/LPS-induced FH^4,19–25^. Nuclear factor-erythroid 2-related factor 2 (NRF2) activation by various drugs protects against hepatic oxidative stress^26,27^ and the related liver injury^28–31^. Proinflammatory cytokines released from macrophage usually induce hepatic inflammation, and TNF-α among these cytokines, could induce liver apoptosis in GalN-pre-sensitized hepatocytes^9,10,16^. Protective autophagy, which could be regulated by AMP-activated protein kinase (AMPK) activation, protects against GalN/LPS-induced liver injury by alleviating apoptosis^22,32^. On the other hand, macrophage activation-induced inflammation also mediates the progression of hepatotoxicity^33,34^, among which NLRP3 inflammasome activation is one most well-documented mechanisms for activating inflammation^30,35^. Accordingly, strategies were proposed to target macrophage/NLRP3 activation for treating liver disease^36,37^.

Traditional herbs have been extensively investigated for treating liver diseases both preclinically and clinically^37–40^. Withaferin A (WA), a natural steroidal lactone isolated from the traditional herb *Withania Somnifera*, possesses various pharmacological activities, such as antitumor, anti-inflammation^41^, and also acts as a leptin sensitizer^42^. WA potently improves acetaminophen-induced hepatotoxicity^43,44^ and diet-induced nonalcoholic steatohepatitis^45^ in rodents. However, whether and how WA improves GalN/LPS-induced FH has not been explored. Mechanically, WA is an NRF2 inducer and WA NRF2-dependently protects against acetaminophen-induced hepatotoxicity^44^. WA also inhibits inflammation by directly inhibiting I*κκ*B activity^46,47^ or NLRP3 inflammasome activation in vitro in immune cells^48,49^, and WA was suggested to AMPKα-dependently protect against cardiovascular disease^50^. Thus, WA probably protects against FH by targeting the macrophage and/or hepatocyte stress via activating NRF2, AMPKα, autophagy, inhibiting I*κκ*B activity or antagonizing the NLRP3 inflammasome activation. In this study, the effects and mechanisms of WA in GalN/LPS-induced FH were investigated, particularly concentrating on the regulation of NLRP3, NRF2, AMPKα, and autophagy signaling.

## Results

### WA attenuated GalN/LPS-induced hepatotoxicity and the inflammatory response

**Fig. 1:**
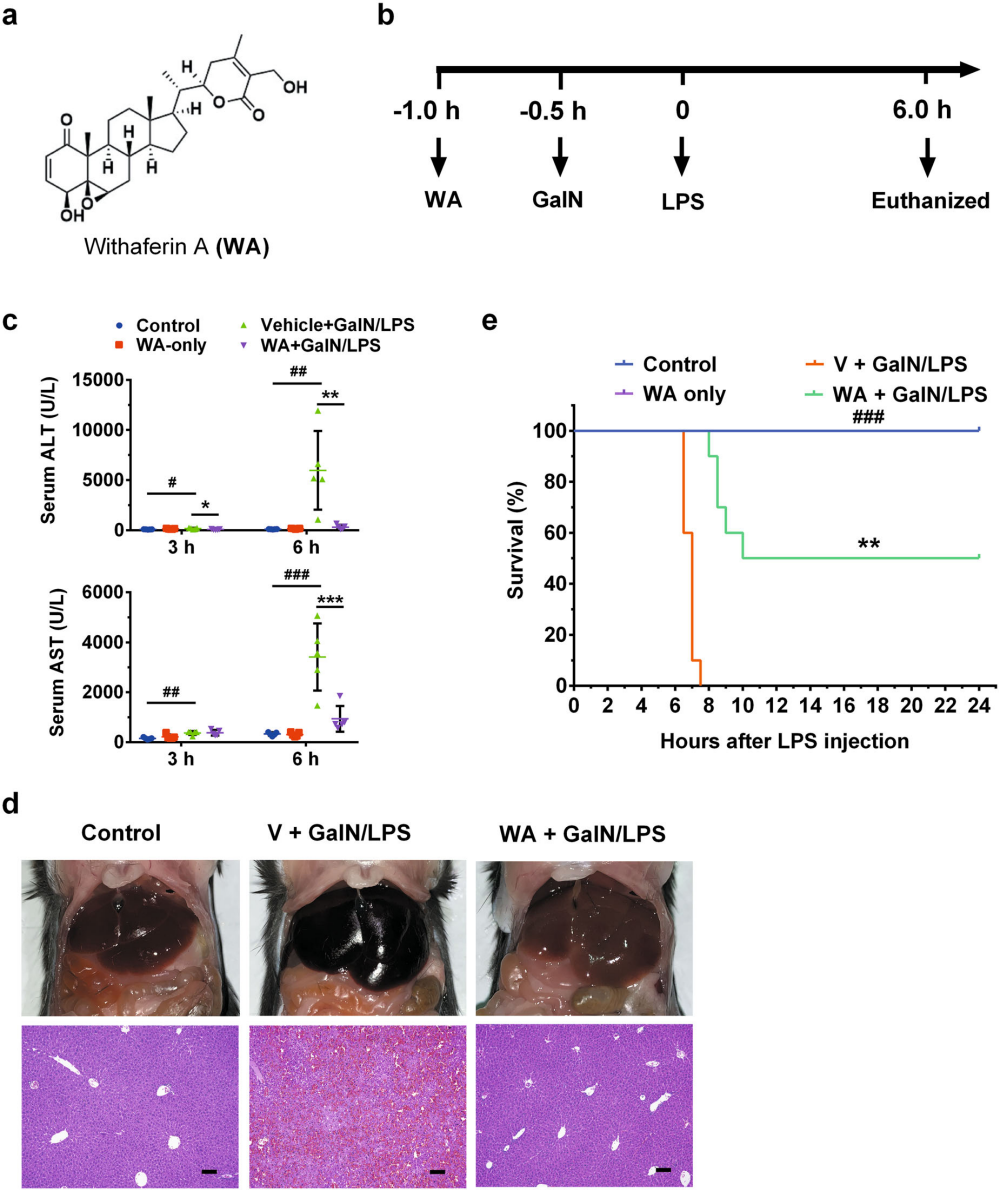
WA protected against GalN/LPS-induced FH in WT mice. **a** The chemical structure of WA. **b** Dosing scheme of animal experiments. **c** Serum ALT and AST levels at 3 h and 6 h after GalN/LSP treatment. **d** Representative liver images and liver histological H&E staining, scale bar 50 μm. **e** Survival curve (*n* = 10). Data are presented as means ± SD (*n* = 5 unless otherwise indicated). Control, mice dosed with control vehicle and saline; WA only, mice dosed with WA and saline; V + GalN/LPS, mice dosed with control vehicle (V), and GalN/LPS; WA + GalN/LPS, mice dosed with WA and GalN/LPS. One-way ANOVA was used for statistical analyses. ^#^*p* < 0.05, ^##^*p* < 0.01, and ^###^*p* < 0.005 versus Control group; *p < 0.05, **p < 0.01, and ***p < 0.005 versus V + GalN/LPS group.

**Fig. 2:**
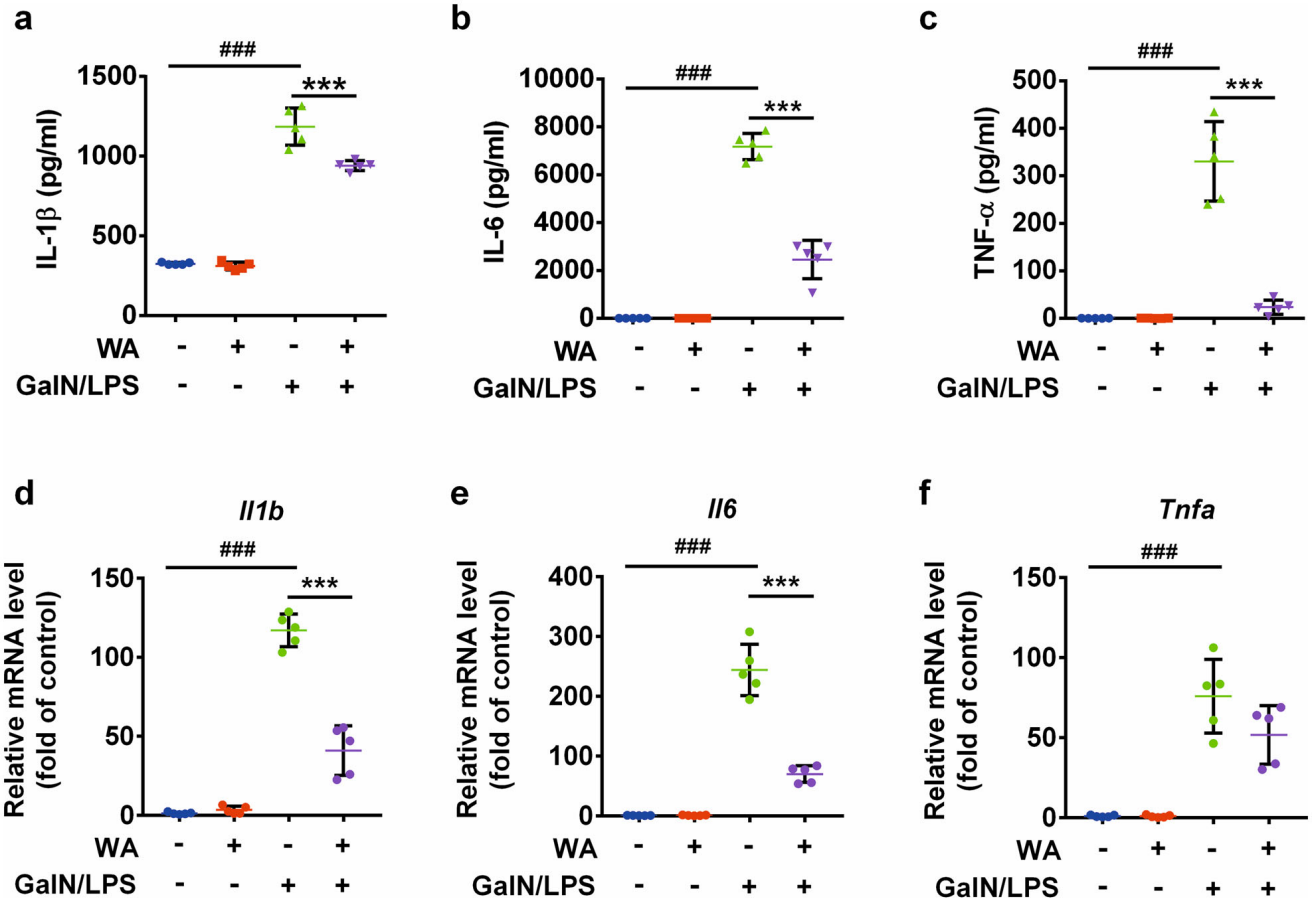
WA suppressed GalN/LPS-induced inflammatory response. Serum IL-1β levels (**a**), Serum IL-6 levels (**b**), and Serum TNF-α levels (**c**). mRNA levels of hepatic *Il1b* (**d**)*, Il6* (**e**), and *Tnfa* (**f**). Data are presented as means ± SD (*n* = 5 unless otherwise indicated). Groups were same as described in Fig. 1 legend. One-way ANOVA was used for statistical analyses. ^###^*p* < 0.005 versus Control group; **p < 0.01 and ***p < 0.005 versus V^+^GalN/LPS group.

To test the preventive effect of WA (structure shown in Fig. 1a) in GalN/LPS-induced FH, mice were pretreated as schemed (Fig. 1b). Serum alanine aminotransferase (ALT) and aspartate aminotransferase (AST) levels were slightly increased at 3 h and markedly increased at 6 h, both of which were decreased by WA (Fig. 1c). WA also sharply attenuated the GalN/LPS-induced histological liver damage, consistent with the normalized gross liver appearance (Fig. 1d). Furthermore, WA significantly improved the survival rate of GalN/LPS-treated mice (Fig. 1e). While FH is accompanied by cytokine release and hepatic inflammation, WA significantly reduced the GalN/LPS-induced increase of serum IL-1β, IL-6, and TNF-α (Fig. 2a–c). WA significantly attenuated the GalN/LPS-induced increase of hepatic *Il1b and Il6* mRNAs, with a tendency to decrease *Tnfa* mRNA (Fig. 2d–f). Thus, WA potently protects against GalN/LPS-induced hepatotoxicity and inflammation.

### WA suppressed hepatic apoptosis in vivo, but not TNF-α-induced hepatocyte death in vitro

**Fig. 3:**
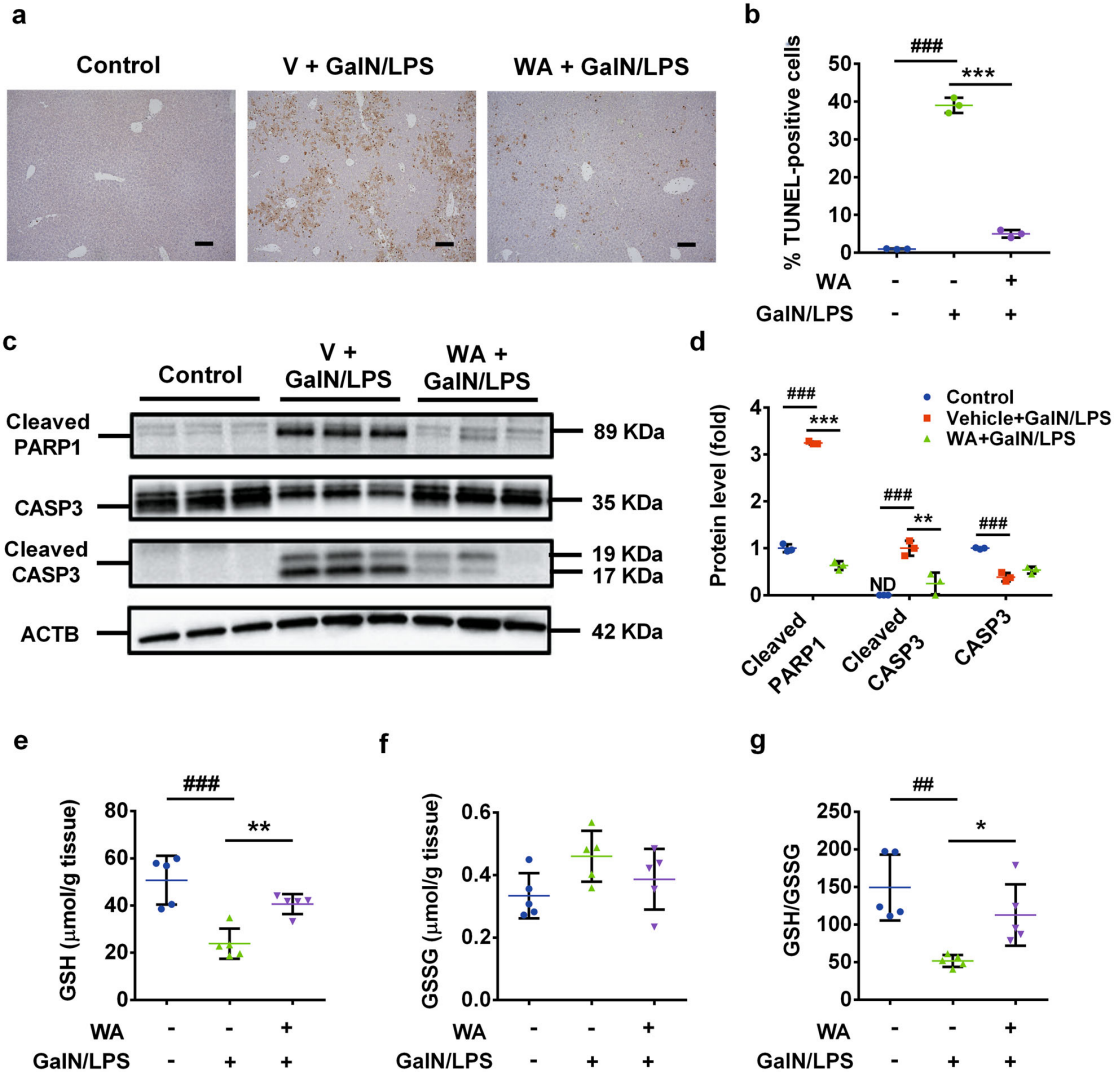
WA suppressed hepatic apoptosis and alleviated hepatic oxidative stress. **a** Representative TUNEL staining of liver sections. **b** Statistical analysis of TUNEL staining (*n* = 3). **c** Western blot analysis of CASP3, cleaved PARP1 and cleaved CASP3 expression. **d** quantitation analyses of cleaved PARP1, CASP3 and cleaved CASP3 (*n* = 3). **e** Hepatic GSH levels. **f** Hepatic GSSG levels. **g** Hepatic GSH/GSSG ratio. Data are presented as means ± SD (*n* = 5 unless otherwise indicated). Groups were the same as described in Fig. 1 legend. One-way ANOVA or unpaired two-tailed *t* test was used for statistical analyses. ^#^*p* < 0.05, ^##^*p* < 0.01, ^###^*p* < 0.005 versus Control group; *p < 0.05, **p < 0.01, ***p < 0.005 versus V + GalN/LPS group.

Typical apoptosis markers, terminal deoxynucleotidyl transferase dUTP nick end labeling (TUNEL) staining, poly-(ADP-ribose) polymerase (PARP1) and caspase 3 (CASP3) cleavage, were next examined to determine whether WA rescued GalN/LPS-induced hepatocyte apoptosis in vivo. WA was found to significantly decrease GalN/LPS-induced positive TUNEL staining (Fig. 3a, b) and attenuate the increase of cleaved CASP3 and cleaved PARP1 (Fig. 3c, d). To test whether WA has a direct anti-apoptosis effect, the ACTD/TNF-α and GalN/TNF-α-induced apoptosis models were employed in primary mouse hepatocytes. As expected, either ACTD or GalN synergized with TNF-α to induce a decrease in cell viability. However, WA pretreatment at 0.01–0.5 μM had no significant effect in reducing ACTD/TNF-α or GalN/TNF-α-induced cell death, while ZVAD efficiently prevented cell death in both models as a positive control (Supplementary Fig. S1). The doses of WA were chosen based on a previous publication^43^ and no toxicity of WA was found at 0.01–0.5 μM (Supplementary Fig. S1). Thus, WA alleviates GalN/LPS-induced apoptotic hepatocyte death in vivo, but shows no direct effect against TNF-α-induced hepatocyte apoptosis in vitro.

### WA alleviated hepatic oxidative stress and rescued the expression of NRF2 target genes in GalN/LPS-treated mice

The effect of WA on oxidative damage was explored. Hepatic glutathione (GSH) levels were significantly depleted by ~50% 6 h after GalN/LPS administration and were recovered to levels comparable with that of control mice by WA treatment (Fig. 3e). While the hepatic levels of oxidized glutathione (GSSG) remained unchanged between groups (Fig. 3f), the GSH/GSSG ratio was significantly decreased and rescued by WA (Fig. 3g), indicative of normalized oxidative stress. Further analyses for how WA affected the expression of NRF2 target gene mRNAs was next performed. In the absence of GalN/LPS treatment, WA significantly upregulated the mRNA levels of several NRF2 target genes in WT mice, and not in *Nrf2*^−/−^ mice (Supplementary Fig. S3a), suggesting an NRF2-dependent effect. GalN/LPS was found to induce NRF2 protein levels when compared with the control group, consistent with a previous study showing a similar role for LPS in inducing NRF2 protein expression although decreasing *Nrf2* mRNA expression^20^. After GalN/LPS administration, WA treatment was found to further increase hepatic NRF2 protein (Supplementary Fig. S2a), and to significantly increase the mRNA levels of *Nrf2* target genes in both WT mice (Supplementary Fig. S2b) and *Nrf2*^−/−^ mice (Supplementary Fig. S3b). Given that NRF2 target gene mRNAs were sharply decreased by GalN/LPS treatment (Supplementary Fig. S2b), WA likely upregulates the mRNA levels of NRF2 target genes in GalN/LPS-treated *Nrf2*^−/−^ mice as a result of its hepatoprotective effect.

### NRF2 was not required for the hepatoprotective effect of WA

WA is known to induce NRF2 signaling and NRF2-dependently alleviates acetaminophen-induced liver injury^43,44^. To further determine if WA protects against FH depending on NRF2, *Nrf2*^−/−^ mice were employed. The results revealed that WA sharply decreased serum ALT levels in GalN/LPS-treated *Nrf2*^−/−^ mice (Supplementary Fig. S2c), and this hepatoprotective effect was confirmed by hematoxylin and eosin (H&E) staining (Supplementary Fig. S2d). Analysis of hepatic *Nrf2* mRNA levels validated the *Nrf2* knockout efficiency of *Nrf2*^−/−^ mice (Supplementary Fig. S3a). These data demonstrate that the presence of NRF2 does not influence the hepatoprotective effect of WA in treating GalN/LPS-induced liver injury, at least under the present experiment conditions.

### WA induced hepatic autophagy in GalN/LPS-treated mice

**Fig. 4:**
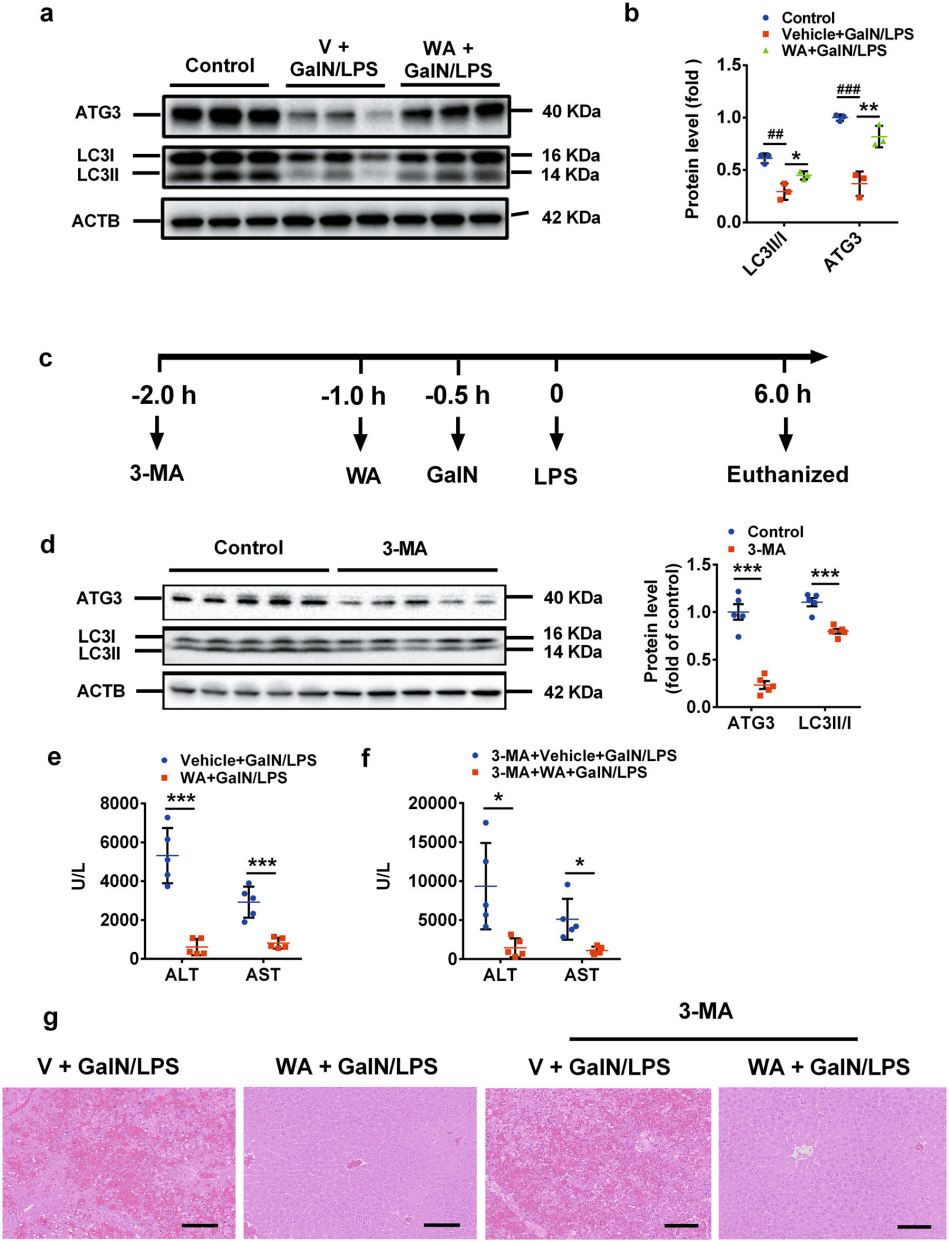
WA induced hepatic autophagy, while 3-MA failed to abolish the hepatoprotective effect of WA. **a** Western blot analysis for ATG3, LC3I, and LC3II (*n* = 3). **b** Quantitation analysis of relative protein levels (*n* = 3). **c** Experimental time scheme. **d** Effect of 3-MA in the expression of ATG3, LC3I, and LC3II. **e** Serum ALT and AST levels in control saline-pretreated mice treated with or without WA. **f** Serum ALT levels and AST levels in 3-MA-pretreated mice treated with or without WA. **g** Representative liver histological H&E staining of control saline-pretreated and 3-MA-pretreated mice treated with or without WA, scale bar 50 μm. Data are presented as means ± SD (*n* = 5 unless otherwise indicated). One-way ANOVA or unpaired two-tailed *t* test was used for statistical analyses. ^##^*p* < 0.01, ^###^*p* < 0.005 versus Control; *p < 0.05, **p < 0.01 and ***p < 0.005 versus V ^+^ GalN/LPS.

Previously, another herbal NRF2 activator, licochalcone A, potently alleviated GalN/LPS-induced liver injury in *Nrf2*^−/−^ mice by inducing autophagy^31^. Thus, whether WA alleviated the FH via induced autophagy similar to licochalcone A was examined. WA significantly rescued the GalN/LPS-induced decrease of autophagy related 3 (ATG3), microtubule-associated protein light chain 3A/B (LC3II), the ratio of LC3II/LC3I (Fig. 4a, b), indicating that WA upregulated hepatic autophagy signaling in GalN/LPS-treated mice.

### Autophagy, hepatocyte AMPKα, or I*κκ*B were not required for the protective effect of WA

3-MA, as a widely-used autophagy inhibitor^51^, was then applied to block WA-induced autophagy. Mice were treated as designed based on previous publications (Fig. 4c)^20,22,31,52,53^. 3-MA at the tested dose, significantly inhibited the expression of ATG3 and decreased the LC3II/LC3I ratio, two typical autophagy markers (Fig. 4d), indicative of the efficient autophagy inhibition by 3-MA. In 3-MA-pretreated mice, WA significantly decreased the GalN/LPS-induced increase of the serum ALT and AST levels to a similar extent with control vehicle-pretreated mice (Fig. 4e, f), consistent with the H&E staining (Fig. 4g). Therefore, 3-MA does not abolish the hepatoprotective effect of WA.

WA was found to protect against myocardial ischemia/reperfusion injury via AMPKα activation^50^, while hepatic AMPKα1 activation protects against GalN/LPS-induced liver injury^22,39^. Whether WA protected against GalN/LPS-induced FH by AMPKα activation was investigated. WA showed no significant effect on expression of AMPKα and p-AMPKα proteins (Supplementary Fig. S4a, b). *Ampka1*^ΔHep^ mice were next employed and analyses of serum ALT levels revealed that the hepatoprotective effect of WA in *Ampka1*^ΔHep^ mice was as potent as in the *Ampka1*^fl/fl^ littermates (Supplementary Fig. S4c), supporting an AMPKα1-independent hepatoprotective effect of WA. Western blot analyses confirmed that the AMPKα1 expression was efficiently depleted in livers of *Ampka1*^ΔHep^ mice (Supplementary Fig. S4d, e). While WA directly targets I*κκ*B to produce its anti-inflammatory effects^46,47^, WA still showed a potent effect in alleviating GalN/LPS-induced liver injury in *Ikkb*^ΔHep^ mice by analyses of serum ALT levels (Supplementary Fig. S4f) and H&E staining (Supplementary Fig. S4g). Thus, the hepatoprotective effect of WA is independent of hepatocyte AMPKα1, hepatocyte I*κκ*B and hepatic autophagy signaling.

### Macrophages depletion abrogated the hepatoprotective effect of WA

**Fig. 5:**
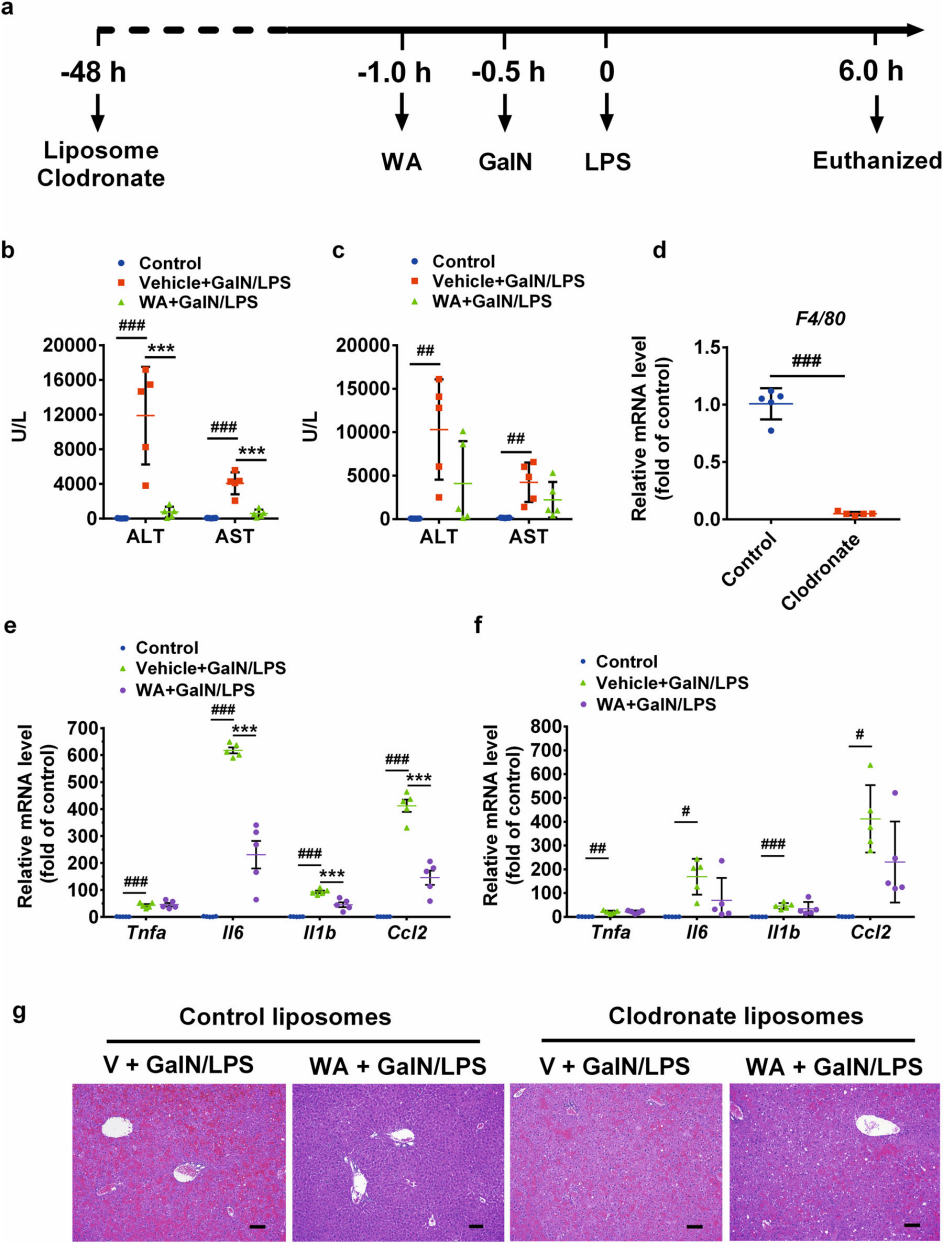
Hepatoprotective effect of WA was abolished in macrophage-depleted mice. **a** Experimental time scheme. **b** Serum ALT and AST levels in control liposomes-pretreated mice. **c** Serum ALT and AST levels clodronate liposomes-pretreated mice. **d** Hepatic *F4/80* mRNA levels of control or clodronate alone-treated mice. **e** Hepatic mRNA levels of proinflammatory cytokines including *Tnfa*, *Il6*, *Il1b*, and *Ccl2* in control liposomes-pretreated mice. **f** Hepatic mRNA levels of inflammation pathway including *Tnfa*, *Il6*, *Il1b*, and *Ccl2* in clodronate liposomes-pretreated mice. **g** Representative images of liver H&E staining, scale bar 50 µm. Data are presented as means ± SD, *n* = 5. Groups were the same as described in Fig. 1 legend. One-way ANOVA was used for statistical analyses. ^#^*p* < 0.05, ^##^*p* < 0.01, and ^###^*p* < 0.005 versus Control group; ***p < 0.005 versus V + GalN/LPS group.

Given that WA still elicited a potent effect in *Nrf2*^−/−^ mice, autophagy-inhibited WT mice, and *Ampka1*^ΔHep^ and *Ikkb*^ΔHep^ mice, the hepatoprotective effect of WA was then suspected to not directly target hepatocytes, but target macrophage, another key determinant of hepatotoxicity. To answer this question, clodronate liposomes were used to deplete macrophage, and mice were treated as schemed (Fig. 5a). In control liposome-injected mice, WA alleviated the GalN/LPS-induced increase of serum ALT and AST levels (Fig. 5b), while upon clodronate injection, WA failed to significantly decrease serum ALT and AST levels (Fig. 5c). Liver *F4/80* mRNA was markedly decreased in clodronate-treated mice compared with control liposome treatment (Fig. 5d), indicative of efficient macrophage depletion by clodronate. Further analyses of liver histology and hepatic proinflammatory cytokines mRNA expression confirmed that the hepatoprotective effect of WA was largely compromised in clodronate-injected mice compared with that in the control liposome-injected mice (Fig. 5e–g). Thus, WA targets macrophage to protect against GalN/LPS-induced FH.

### WA prevented GalN/LPS-induced FH partially by inhibiting activation of the NLRP3 inflammasome

**Fig. 6:**
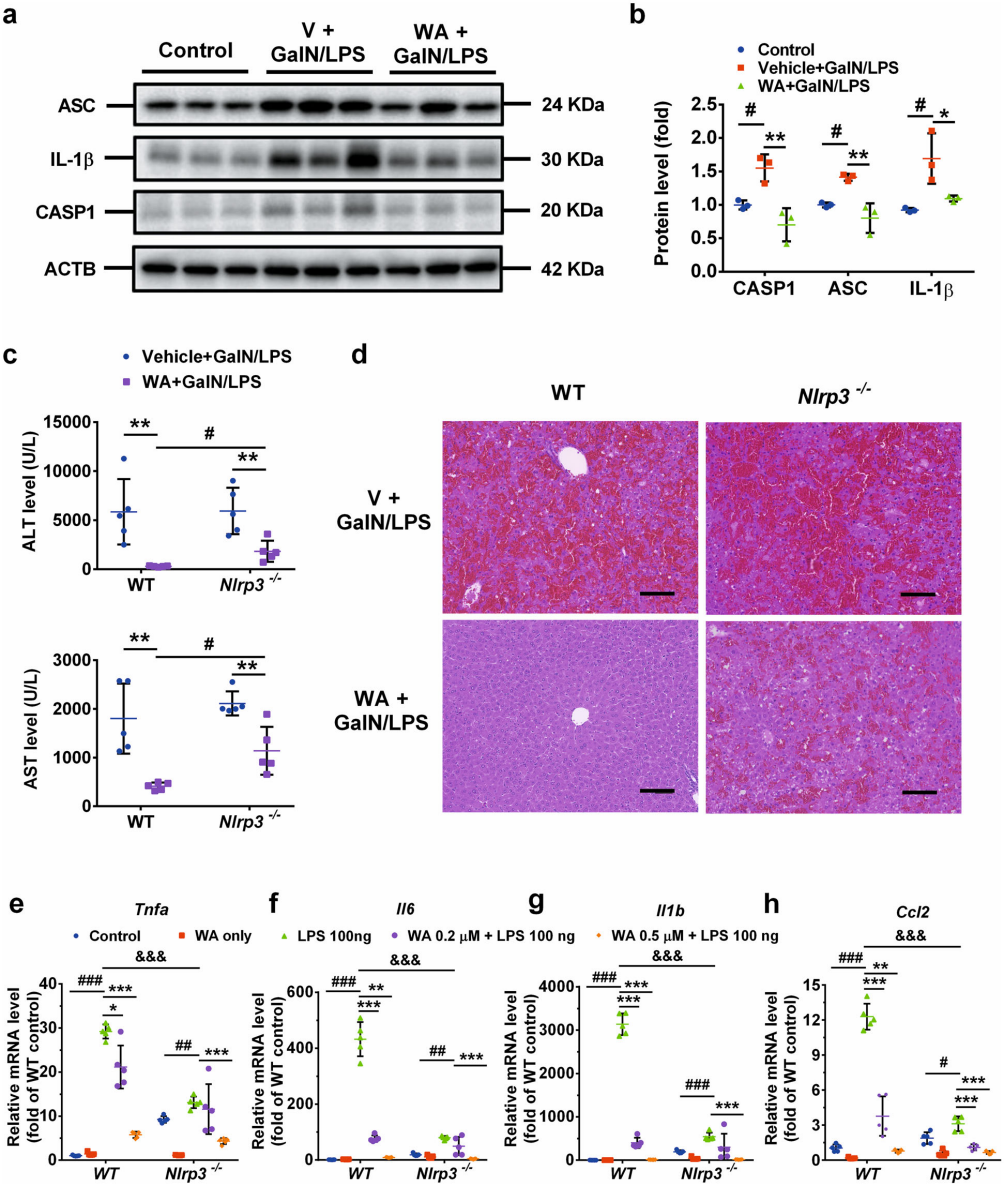
WA attenuated the hepatic inflammasome activation in WT mice, while the hepatoprotective effect of WA was partially compromised in *Nlrp3*^−/−^ mice. **a** Representative images of western blot analyses for CASP1, ASC and IL-1β in WT livers (*n* = 3). **b** Quantitation for CASP1, ASC and IL-1β (*n* = 3). **c** Serum ALT and AST levels in GalN/LPS-treated WT or *Nlrp3*^−/−^ mice treated with or without WA. **d** Representative hepatic H&E staining of V or WA-treated WT or *Nlrp3*^−/−^ mice subjected to GalN/LPS challenge, scale bar 50 µm. Effect of WA in mRNA levels of *Tnfa* (**e**), *Il6* (**f**), *Il1b* (**g**), and *Ccl2* (**h**), in LPS-treated WT and *Nlrp3*^−/−^ macrophage. One-way ANOVA or two-tailed t test was used for statistical analyses. Data were presented as mean ± SD (*n* = 5 unless otherwise indicated). For the animal study, ^#^*p* < 0.05 versus Control; *p < 0.05 or ***p < 0.005 versus V+GalN/LPS. For the macrophage culture study, ^#^*p* < 0.05, ^##^*p* < 0.01, or ^###^*p* < 0.005 versus WT Control or *Nlrp3*^−/−^ Control, respectively; *p < 0.05, **p < 0.01, or ***p < 0.005 versus WT LPS or *Nlrp3*^−/−^ LPS, respectively. ^&&&^*p* < 0.005 represents comparison between WT LPS and *Nlrp3*^−/−^ LPS group.

NLRP3 inflammasome activation is a well-documented pathway for inflammation induction in macrophage in vitro and in various diseases models in rodents^30,35,54,55^, while WA directly antagonizes NLRP3 inflammasome activation in vitro^48,49^. Thus, the question arose whether WA attenuated GalN/LPS-induced FH by inhibiting NLRP3 inflammasome activation. The time course of NLRP3 inflammasome-mediated inflammation signaling activation was examined, and western blot analyses demonstrated that interleukin 1β (IL-1β) was significantly induced as early as 1 h, which peaked 3 h after LPS dosing, and the protein levels of both NLRP3 and ASC were significantly upregulated and peaked 6 h post LPS dosing (Supplementary Fig. S5a, b). The time-course change of NLRP3 inflammasome signaling correlated well with the time-course change of serum ALT and AST levels, both of which were also sharply upregulated 6 h post LPS dosing, while only showing a slight increase 3 h post LPS dosing (Fig. 1c). Given that all tested proteins involved in NLRP3 inflammasome signaling were consistently upregulated 6 h post LPS dosing, how WA affected the hepatic NLRP3 inflammasome signaling activation in GalN/LPS-dosed mice was next investigated. WA significantly attenuated the GalN/LPS-induced increase of the proteins including ASC, cleaved-CASP1 and IL-1β in liver, all of which play a key role in NLRP3 inflammasome activation (Fig. 6a, b). To examine whether the inhibitory effect of WA in hepatic NLRP3 inflammasome activation is a cause or result of its hepatoprotective effect, *Nlrp3*^−/−^ mice were employed. Analyses of serum ALT and AST levels showed that *Nlrp3* deficiency did not abrogate, but significantly reduced the hepatoprotective effect of WA by ~20% percent (Fig. 6c), which were further supported by histology analyses (Fig. 6d). Thus, WA alleviated GalN/LPS-induced FH partially dependent on NLRP3.

To test whether the direct anti-inflammatory role of WA depends on NLRP3, primary macrophages were isolated from WT mice and matched *Nlrp3*^−/−^ mice. The knockout efficacy of *Nlrp3* was validated (Supplementary Fig. S5c). In WT macrophage, WA, at 0.2 µM and 0.5 µM, dose-dependently attenuated LPS-induced *Tnfa*, *Il6*, *Il1b* mRNAs (Fig. 5e–g) and *Nlrp3* mRNA (Supplementary Fig. S5d). The inhibitory effects of WA in LPS-induced upregulation of *Tnfa*, *Il6*, *Il1b*, were lost in *Nlrp3*^−/−^ macrophage when WA was used at the lower dose of 0.2 µM, while this effect was still found with WA at the higher dose of 0.5 µM in *Nlrp3*^−/−^ macrophage (Fig. 6e–g), suggesting that WA has both NLRP3-dependent and NLRP3-independent anti-inflammatory effects. Consistent with the possibility that WA has an NLRP3-independent anti-inflammatory effect, LPS-induced upregulation of chemokine (C-C motif) ligand 2 *(Ccl2)* mRNA, which does not act directly downstream of NLRP3, was dose-dependently decreased by WA at 0.2 µM and 0.5 µM in both WT macrophage and *Nlrp3*^−/−^ macrophage (Fig. 6h). Notably, LPS-induced upregulation of *Tnfa*, *Il6*, *Il1b* and *Ccl2* mRNAs were all markedly decreased in *Nlrp3*^−/−^ macrophage compared with WT macrophage (Fig. 6e–h), indicating a key role for NLRP3 in mediating LPS-induced inflammation. WA at 0.2 µM and 0.5 µM was nontoxic to both WT and *Nlrp3*^−/−^ macrophage (Supplementary Fig. S5e, f). These data suggest that WA produces a strong anti-inflammatory effect in macrophages, which is only partially dependent on NLRP3.

### WA therapeutically improved GalN/LPS-induced hepatotoxicity

**Fig. 7:**
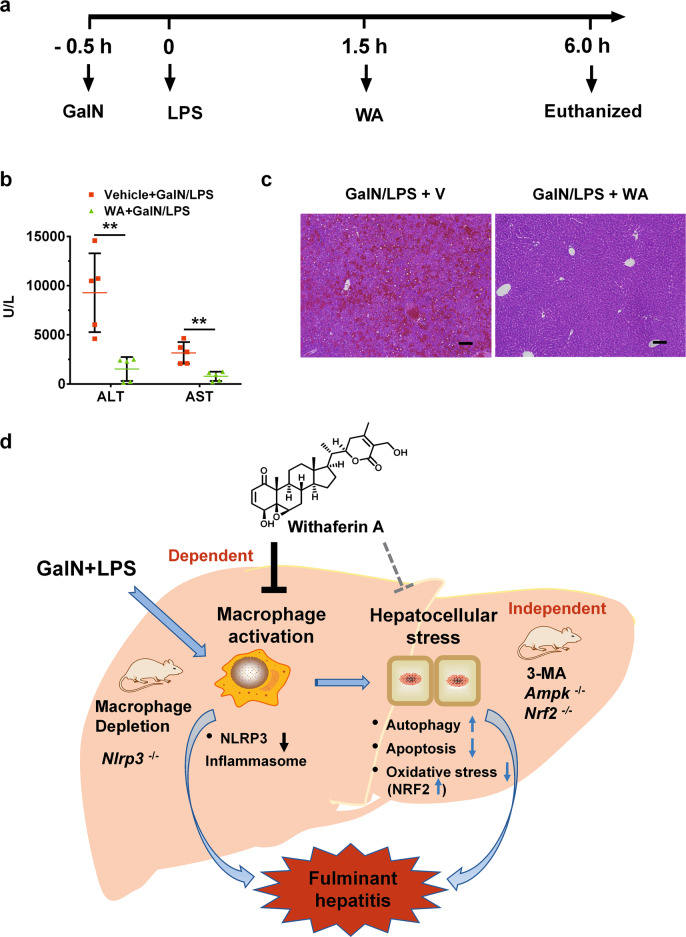
WA therapeutically alleviated GalN/LPS-induced liver injury. **a** Experimental time scheme. **b** Serum ALT and AST levels in GalN/LPS-treated mice followed by vehicle (V) or WA treatment. **c** Representative hepatic H&E staining of V or WA-treated WT mice subjected to GalN/LPS challenge, scale bar 50 µm. **d** graphic abstract for major findings and proposed mechanism. Unpaired two-tailed Student’s *t* test was used for statistical analyses. GalN/LPS + V, mice treated with GalN/LPS followed by vehicle treatment; GalN/LPS + WA, mice treated with GalN/LPS followed by WA treatment. Data were presented as mean ± SD (*n* = 5). **p < 0.01 versus GalN/LPS + V group.

To further explore the therapeutic potential of WA in GalN/LPS-induced hepatotoxicity, a therapeutic dosing scheme was designed to treat WT mice (Fig. 7a). Notably, WA was found to markedly alleviate the GalN/LPS-induced increase of serum ALT and AST levels as well as histological damage even when WA was dosed 2 h after GalN dosing compared with control vehicle (Fig. 7b, c). These data support the view that WA has a potent therapeutic effect in GalN/LPS-induced liver injury in mice.

## Discussion

FH, also known as acute liver failure, could lead to lethality with liver transplantation as the only curable treatment^1,2^. Thus, pharmacotherapeutic discoveries are needed to treat this disease. WA was reported as a hepatoprotective agent in acetaminophen-induced hepatotoxicity mainly due to hepatocyte necrosis^43,44^, and diet-induced nonalcoholic steatohepatitis^45^ that is characterized by hepatic steatosis, inflammation and fibrosis, while little is known about its role and mechanism in alleviating GalN/LPS-induced FH that is associated with apoptotic hepatocyte death and inflammation.

In the present study, hepatocellular stress alleviation that could be mediated by rescuing expression of hepatic NRF2 target genes, hepatic I*κκ*B inhibition, and protective autophagy induction via AMPKα signaling were first excluded as contributors to the hepatoprotective effects of WA by using *Nrf2*^−/−^ mice, *Ikkb*^ΔHep^ mice, *Ampka1*^ΔHep^ mice and 3-MA-injected WT mice under the conditions used in this study. The hepatoprotective properties of WA is likely due to effects on macrophage rather than by directly protecting hepatocytes. The protective effects of WA was sharply deminished by macrophage depletion, supporting the view that WA targets the macrophage to treat experimental FH. Further studies demonstrated that the protective effect of WA against the GalN/LPS-induced FH was partially dependent on NLRP3 inflammasome antagonism in vivo and has a direct anti-inflammatory influence partially dependent on the presence of NLRP3 in macrophage in vitro, indicating that other targets, beyond NLRP3, mediate the anti-inflammatory effect of WA. WA inhibits inflammation by direct hyperphosphorylation of I*κκ*B to inhibit the I*κκ*B activity^46,47^. While the possibility that hepatocyte I*κκ*B plays a role in the hepatoprotective effect of WA on GalN/LPS-induced FH was excluded by using *Ikkb*^ΔHep^ mice, it is still possible that WA targets the I*κκ*B present in macrophage to produce its anti-inflammatory effects. Thus, molecular targets present in macrophage other than NLRP3, such as I*κκ*B, that mediate the anti-inflammatory effect of WA deserve further study.

Previously, WA was found to alleviate acetaminophen-induced liver injury dependent on NRF2 induction^44^. In contrast, the hepatoprotective effect of WA was currently found to be independent of NRF2 in the GalN/LPS model as revealed by using *Nrf2*^−/−^ mice. This may be due to the different pathological hepatotoxicity mechanisms for these two liver injury models, where acetaminophen mainly induces hepatocyte necrosis^56^ and GalN/LPS mainly initiates hepatocyte apoptosis^15^. More importantly, the roles of NRF2 deficiency in these two models are different. *Nrf2*^−/−^ mice were protected from GalN/LPS-induced FH^31^, but showed an enhanced hepatotoxicity in acetaminophen-treated mice^57^. Consistent with these findings, another herbal NRF2 activator, licochalcone A, also showed a potent hepatoprotective effect in GalN/LPS-treated *Nrf2*^−/−^ mice^31^. This same study demonstrated that the autophagy inhibitor 3-MA did not abolish the hepatoprotective effect of licochalcone A^31^. Similarly, the current study found that 3-MA did not abolish the hepatoprotective effect of WA. These results suggest that the NRF2 presence and autophagy induction play only minor roles in mediating the hepatoprotective effects of these herbs at the employed experiment conditions. Thus, WA targets macrophage instead of directly protecting hepatocyte stress pathways to alleviate GalN/LPS-induced FH, at least under the present experiment conditions.

Evidence in the current study reveal that the macrophage-NLRP3 axis at least partially determines the hepatoprotective effect of WA. Indeed, previous studies also support the concept of targeting macrophage to alleviate liver injury^23,33,34,36,37,58–60^ and that pharmacotherapies alleviate GalN/LPS-induced liver injury via inhibiting NLRP3 inflammasome activation^30,61^. Both macrophage and NLRP3 have pleotropic effects in maintaining systemic homeostasis. For example, macrophage are divided into two polarized types, the M2-like macrophage that are hepatoprotective and the M1-like macrophage that stimulate inflammation^62^. NLRP3 is physiologically beneficial in maintaining metabolic and immune homeostasis but could be detrimental upon overactivation^63,64^. In the current study, the hepatoprotective effect of WA on GalN/LPS-induced FH depends on the presence of macrophage and partially dependent on NLRP3, which may be explained by the potent inhibitory effects of WA on the adverse events caused by overactivation of the macrophage-NLRP3 axis in the GalN/LPS model. However, no statistically significant difference in sensitivity to GalN/LPS-induced FH was found between *Nlrp3*^-/-^ mice and the background strain-matched WT mice, as well as between macrophage-depleted mice and vehicle-treated control mice, which could result from a pooled effect of both the diminished beneficial effects and harmful effects upon loss of macrophage or the *Nlrp3* gene, or due to the potent toxic effects of GalN/LPS that overwhelm the potential influence of macrophage and NLRP3 on the disease phenotype. These results suggest that macrophage could have different effects between modulating the onset of the disease and WA suppression of the disease. In line with this point, inflammasome-deficient mice have increased severity to induced nonalcoholic steatohepatitis^65^, and NLRP3 inflammasome overactivation is required for fibrosis development and thus antagonism of NLRP3 inflammasome activation reduces nonalcoholic fatty liver disease^66,67^.

In summary, the current study demonstrates a novel role for WA in both preventing and therapeutically alleviating GalN/LPS-induced FH via targeting the macrophage that is partially dependent on NLRP3 (Fig. 7d). WA, although having effects of inducing hepatic NRF2 and autophagy signaling, the dependence of its hepatoprotective effect in these two signaling pathways is minor under the experimental conditions used in this study. These results demonstrate that WA targets macrophages to treat GalN/LPS-induced FH, which further support the possibility of targeting hepatic macrophage to treat liver disease^36^ and support the potential of WA to be repurposed as a hepatoprotective agent against the clinical FH, particularly featured with TNF-α-related apoptosis.

## Methods and Materials

### Reagents

WA was purchased from ChromaDex (Irvine, CA, USA). ACTD, GalN, LPS, 3-methyladenine (3-MA), dimethyl sulfoxide, N-benzyloxycarbonyl-Val-Ala-Asp(O-Me) fluoromethyl ketone (ZVAD) and other reagents, if not otherwise indicated, were purchased from Sigma Aldrich (St. Louis, MO, USA). Recombinant mouse TNF-α was from Peprotech (Rocky Hill, NJ, USA). Cell-counting kit 8 was from Dojindo Molecular Technologies Inc (Rockville, MD, USA). ALT and AST kits were from Catachem (Oxford, CT, USA). Clodronate liposomes (neutral) were obtained from FormuMax (Sunnyvale, CA, USA). Antibodies against CASP3, cleaved PARP1, LC3I/II, ATG3, ASC, IL-1β, AMPKα, p-AMPKα, NRF2 and β-actin (ACTB) were from Cell Signaling Technology (Danvers, MA, USA). CASP1 antibody was from Santa Cruz Biotechnology (Dallas, Texas, USA). Details for the antibodies are described in Supplementary Table S1.

### Animal Experiments

Wild-type (WT) mice, *Nrf2*^−*/*−^, *Nlrp3*^−*/*−^, or *Ampka1*^fl/fl^ mice on a C57BL/6J background were purchased from the Jackson Laboratory, while *Ikkb*^ΔHep^ mice on the C57BL/6J background were described previously^68,69^. All mice were housed in the National Cancer Institute animal facility that was a pathogen-free environment controlled for temperature, light (25 °C, 12-h light/dark cycle) and humidity (45–65%). Albumin-Cre mice as described previously^69^ were bred with *Ampka1*^fl/fl^ mice to generate *Ampka1*^ΔHep^ mice. Age and body weight-matched 6- to 8-week-old males were randomized into groups (at least *N* = 5) and treated with 700 mg/kg of GalN and 50 µg/kg of LPS or other reagents as described in detail in the Supplementary materials. The National Cancer Institute Animal Care and Use Committee approved all animal experiments conducted in this study.

### Cell culture

Primary hepatocytes, isolated as described previously^70^, were pretreated with WA at 0, 0.01, 0.1, and 0.5 μM or 10 μM ZVAD for 30 min, followed by 5 mM of GalN or 0.3 μM ACTD for 30 min, and then 25 ng/mL of recombinant mouse TNF-α for additional 13 h. Cell counting kit-8 reagent was used to measure cell viability at 13 h after TNF-α treatment. Dimethyl sulfoxide (0.1%) was used as control vehicle. Primary peritoneal macrophage were isolated as described previously with minor modifications^54^. The macrophage were pretreated with WA at 0, 0.2, and 0.5 µM for 2 h, followed by 100 ng/mL of LPS treatment for additional 18 h. Other details can be found in Supplementary Materials.

### Blinding, randomization, statistical analysis, and reproducibility of experiments

The investigators were blind to the experiment treatments. The experiments were repeated at least twice. The animal experiments were randomized. Statistical analyses were determined by two-tailed unpaired student’s *t* test between two groups or by one-way ANOVA followed by Dunnett’s multiple comparisons test among multiple groups for independent samples using GraphPad Prism 7.0 (San Diego, CA, USA). To predetermine sample sizes, power analysis was performed using StatMate version 2.0 (GraphPad Software). All data were presented as mean ± SD. *P* values < 0.05 were considered statistically significant.

## Supplementary information

Supplementary Methods

Legends to Supplementary Figures

Supplementary Fig. S1

Supplementary Fig. S2

Supplementary Fig. S3

Supplementary Fig. S4

Supplementary Fig. S5

Supplementary Table 1

Supplementary Table 2
