# Supplementary material for: Withaferin A alleviates fulminant hepatitis by targeting macrophage and NLRP3
Source: Cell Death Dis. 2021 Feb 11;12(2):174. doi: 10.1038/s41419-020-03243-w (PMC7878893; doi:10.1038/s41419-020-03243-w)
Supplement: Supplementary file 1 — Supplementary Methods [file 41419_2020_3243_MOESM1_ESM.pdf]

Supplementary Methods

Animal Treatments

To test the preventative effects of WA in treating GalN/LPS-induced liver injury, body weight and age-matched 6- to 8-week-old, 20-25 g mice of each mouse strain (WT, *Nrf2*$^{-/-}$, *Nlrp3*$^{-/-}$ and *Ampka1*$^{\Delta Hep}$) were randomly divided into four groups (Control group, WA only group, Vehicle + GalN/LPS group, and WA + GalN/LPS group) or two groups (Vehicle + GalN/LPS group, and WA + GalN/LPS group) as indicated in each experiment. In brief, the same volume (200 µL/20 g mice) of saline containing 5% dimethyl sulfoxide and 5% Tween 80 or WA (10 mg/kg dissolved in 5% dimethyl sulfoxide and 5% Tween 80-contained saline) was administered intraperitoneally to mice, followed by treatment with GalN (700 mg/kg, dissolved in saline, *i.p.*) 30 min after WA dosing, and then injected with LPS (50 µg/kg, dissolved in saline, *i.p.*) 30 min after GalN dosing. Serum were collected 3 h or 6 h after LPS administration, and mice killed by CO_2 inhalation to collect blood via the orbital vein and livers for further analysis 6 h after LPS dosing.

To test the effect of WA in the survival rate of GalN/LPS-treated mice, the mice (n=10) treated with WA once followed by GalN/LPS treatment as described above. Mice with either a combined score of 20 as described[1] or a body temperature of no more than 30°C were immediately killed per the animal protocol approved by the National Cancer Institute Animal Care and Use Committee.

To test if the preventative effects of WA in treating GalN/LPS-induced liver injury depended on autophagy signaling, mice were intraperitoneally injected with 3-MA (30 mg/kg) for 2 h prior to LPS dosing, followed by dosing of WA (10 mg/kg) and GalN/LPS (700 mg/kg/50 µg/kg) as described above. For the macrophage depletion study, age and weight-matched WT mice (20-25 g) were treated with clodronate liposomes (200 µL/mice) or control liposomes via intraperitoneally injection and 48 h later, the mice were subjected to WA/control vehicle treatment followed by GalN/LPS dosing as described above. All mice were killed 6 h after LPS dosing by CO_2 inhalation to collect blood via orbital vein and livers for study.

To test whether WA has a therapeutic effect in treating GalN/LPS-induced liver injury, 8-week-old body weight-matched C57BL/6J mice were randomly divided into two groups, control vehicle group and WA-treated group. Mice were treated with GalN/LPS as described above, while WA (10 mg/kg) or control vehicle were intraperitoneally injected at 2 hours after GalN dosing. All mice were killed by CO_2 inhalation 6 h after LPS dosing to collect serum and tissues for further analyses.

Histopathological Evaluation

Formalin-fixed liver tissues were embedded in paraffin and five µm thick sections were cut for both H&E and TUNEL staining. Further sample processing for H&E staining, and TUNEL staining were performed at VitroVivo Biotech (Rockville, MD, USA). The histological changes of H&E-stained slices were evaluated in randomly chosen histological fields using light microscopy. The number of apoptotic cells of TUNEL-stained slices was recorded at 400 times magnification. At least three tissue sections in each group were analyzed.

Biochemical Analysis of Serum and Liver

Biochemical parameters including levels of serum ALT and AST (diluted 5 fold with saline), as well as levels of glutathione and oxidized glutathione in serum or liver, were measured using commercial kits based on the manufacturer's instructions. PeroxiDetect kit, superoxide dismutase kit, and glutathione assay kit were from Sigma Aldrich.

Serum Enzyme-linked Immunosorbent Assay

Enzyme-linked immunosorbent assay kits for mouse TNF-α, IL-6 and IL-1β were from Sigma Aldrich. Fifty µL of serum was used for detecting the levels of TNF-α, IL-1β, and IL-6 using each assay kit. Serum from Vehicle or WA-treated GalN/LPS-treated mice were diluted 5 fold prior to analysis. All other procedures were in accordance to respective manufacturers manual.

Primary Hepatocyte Cultures

Primary hepatocytes were isolated from C57BL/6J mice and seeded in 96-well (5×10^4 cells/well) or 12-well (3×10^5 cells/well) plates at 90% confluency. The hepatocytes were incubated in Williams Medium E (Gibco, Gaithersburg, MD, USA) containing 10% fatal bovine serum (Sigma Aldrich) and 1% antibiotics (equal mixture of 0.5% penicillin and 0.5% streptomycin), 1% insulin-transferrin-selenium and 400 ng/mL dexamethasone overnight before treatment. All cells were maintained at 37°C in a 5% CO_2/air environment.

Primary Macrophage Culture

Three mL of 3% brewer thioglycolate medium (REF 211716, BD bioscience, San Jose, CA, USA) was intraperitoneally injected per mouse, allowing the inflammatory response to proceed for three days. Mice were killed by cervical dislocation and soaked with 75% alcohol. Ten mL of ice-cold phosphate-buffered saline (containing 5% bovine serum albumin) were intraperitoneally injected into each mouse and the abdominal skin was rubbed for 5 min to thoroughly mix the injected phosphate-buffered saline with the peritoneal macrophage cells. Then fluid was aspirated from the peritoneum by using a 10 mL syringe with 23-G needle packaged in a soft shell outside of the needle to avoid sharp needle-mediated bleeding. The peritoneal exudate cells were centrifuged for 10 min at 400g, 4°C. Macrophage were seeded at 70-90% confluency in 12-well plates in RIPM1640 medium (Corning, Manassas, VA, USA) containing 10% fatal bovine serum and 1% antibiotics (equal mixture of 0.5% penicillin and 0.5% streptomycin), cultured in the plates for 4 hours to allow the macrophage to attach to the bottom of the culture dish, and then twice washed with phosphate-buffered saline.

Quantitative Real-time Polymerase Chain Reaction Analysis

The livers were flash frozen in liquid nitrogen and stored at -80°C. Total RNA from frozen livers was extracted with TRIzol reagent (Invitrogen, Carlsbad, CA). cDNA was synthesized from 1 µg of total RNA using qScript cDNA SuperMix (Gaithersburg, MD). Analysis was performed by using the ABI PRISM 7900 Sequence Detection System (Applied Biosystems, Bedford, MA). Values were normalized to *Actb* mRNAs and the

results expressed as fold change relative to the control group. Primer sequences are provided in the Supplementary Table 2.

Western Blot Analysis

Liver proteins were prepared in RIPA lysis buffer with protease inhibitors and phosphatase inhibitors cocktail. Then SDS-PAGE was conducted, and bands were transferred to PVDF membrane. The membrane was blocked with 5% (w/v) bovine serum albumin for 2 h and then incubated with primary antibody and secondary antibody, both of which were dissolved in 5% bovine serum albumin. The membranes were visualized, and signals obtained were normalized to ACTB. To quantitate the western blot bands, the blots were analyzed using ImageJ software (National Institutes of Health, Bethesda, MD).

Reference:

1. Ziegler, T. *et al.* Angiopoietin 2 mediates microvascular and hemodynamic alterations in sepsis. *J. Clin. Invest.* 123, 3436-3445 (2013).
