# Supplementary material for: Withaferin A alleviates fulminant hepatitis by targeting macrophage and NLRP3
Source: Cell Death Dis. 2021 Feb 11;12(2):174. doi: 10.1038/s41419-020-03243-w (PMC7878893; doi:10.1038/s41419-020-03243-w)
Supplement: Supplementary file 2 — Legends to Supplementary Figures [file 41419_2020_3243_MOESM2_ESM.pdf]

Legends to supplementary figures

Supplementary Fig. S1. Effect of WA in the cell viability of ATCD/TNF-α or GalN/TNF-α-treated primary hepatocyte *in vitro*. a, cell viability of hepatocytes treated with WA alone at the doses of 0-0.5 μM (n=5). b and c, cell viability of ACTD/TNFα- (b) or GalN/TNF-α (c)-treated hepatocytes treated with WA at the doses of 0, 0.01, 0.1, 0.5 μM or ZVAD at 10 μM as a positive control, n=6. Data are presented as means ± SD. One-way ANOVA was used for statistical analyses. $^{###}p < 0.005$; $^{***}p < 0.05$, $^{***}p < 0.005$. DMSO, dimethyl sulfoxide.

Supplementary Fig. S2. WA induced hepatic NRF2 signaling, while the hepatoprotective effect of WA was independent of NRF2. a, NRF2 protein levels in mouse livers. b, mRNA levels of *Nrf2* and its target genes in mouse livers. c and d, serum ALT levels and liver H&E staining in GalN/LPS-treated *Nrf2*$^{-/-}$ mice (n=7). Data are presented as means ± SD (n=5 unless otherwise indicated). Groups were same as described in Figure 1 legend. One-way ANOVA or unpaired two-tailed t test was used for statistical analyses. $^{\#}p < 0.05$, $^{\#\#}p < 0.01$, $^{###}p < 0.005$ versus Control group; $^{*}p < 0.05$, $^{**}p < 0.01$, $^{***}p < 0.005$ versus V + GalN/LPS group.

Supplementary Fig. S3: Effects of WA on NRF2 target genes in non-treated WT and *Nrf2*$^{-/-}$ mice, or in GalN/LPS-treated *Nrf2*$^{-/-}$ mice. a, hepatic mRNA levels of *Nrf2* and its target genes in normal non-challenged mice treated with control vehicle (Control) or WA at doses of 10 mg/kg, n=5. b, hepatic *Nrf2* mRNA levels and NRF2 target genes in GalN/LPS-challenged *Nrf2*-null mice treated with control vehicle (V + GalN/LPS) or 10 mg/kg of WA (WA + GalN/LPS), n=8. Data are presented as means ± SD. Two-tailed t test was used for statistical analyses. $^{*}p < 0.05$ and $^{***}p < 0.005$ versus WT: Control group for Fig. S3a; and $^{***}p < 0.005$ versus V + GalN/LPS group for Fig.S3b.

Supplementary Fig. S4: The hepatoprotective effect of WA was independent of hepatic AMPKα and Iκκ B. a, western blot analysis for AMPKα, and p-AMPKα (n=3). b, quantitation analysis for relative protein levels (n=3). c, serum ALT levels for GalN/LPS-treated *Ampka1*$^{\Delta Hep}$ mice and its matched *Ampka1*$^{fl/fl}$ mice treated with or

without WA. d, hepatic protein levels of AMPKα in the livers of *Ampka1*$^{\Delta Hep}$ and its matched *Ampka1*$^{fl/fl}$ mice (n=3). e, quantitation analysis for AMPKα levels (n=3). f, serum ALT levels for GalN/LPS-treated *Ikkb*$^{\Delta Hep}$ mice and its matched *Ikkb*$^{fl/fl}$ mice treated with or without WA. g, liver H&E staining for the GalN/LPS-treated *Ikkb*$^{\Delta Hep}$ mice or matched *Ikkb*$^{fl/fl}$ that were treated with or without WA. Data are presented as means ± SD (n=5 unless otherwise indicated). One-way ANOVA or unpaired two-tailed t test was used for statistical analyses. $^{\#\#}p < 0.01$, $^{\#\#\#}p < 0.005$ versus Control; $^{*}P<0.05$, $^{**}P<0.01$ and $^{***}p < 0.005$ versus V + GalN/LPS.

Supplementary Fig. S5: Analyses of primary macrophage. a, western blot analysis for ASC, cleaved-CASP1 and IL-1β for GalN/LPS treated mice at 0 h, 1 h, 3 h and 6 h post LPS dosing. b, quantitation analysis for relative protein levels (n=4). c, mRNA levels of macrophage isolated from WT mice and *Nlrp3*$^{-/-}$ mice. d, effects of WA at doses of 0, 0.2, 0.5 μM in LPS-induced upregulation of *Nlrp3* mRNA in primary peritoneal macrophage isolated from WT mice. e and f, the effect of testing reagents in the cell viability when used alone in WT macrophages (e) and *Nlrp3*$^{-/-}$ macrophages (f). Data are presented as means ± SD (n =5). One-way ANOVA or two-tailed t test was used for statistical analyses. Groups was same as described in Fig.6. $^{\#\#\#}p < 0.005$ versus Control group; $^{**}p < 0.01$.
