# Supplementary figures and images for: Withaferin A alleviates fulminant hepatitis by targeting macrophage and NLRP3
Source: Cell Death Dis. 2021 Feb 11;12(2):174. doi: 10.1038/s41419-020-03243-w (PMC7878893; doi:10.1038/s41419-020-03243-w)

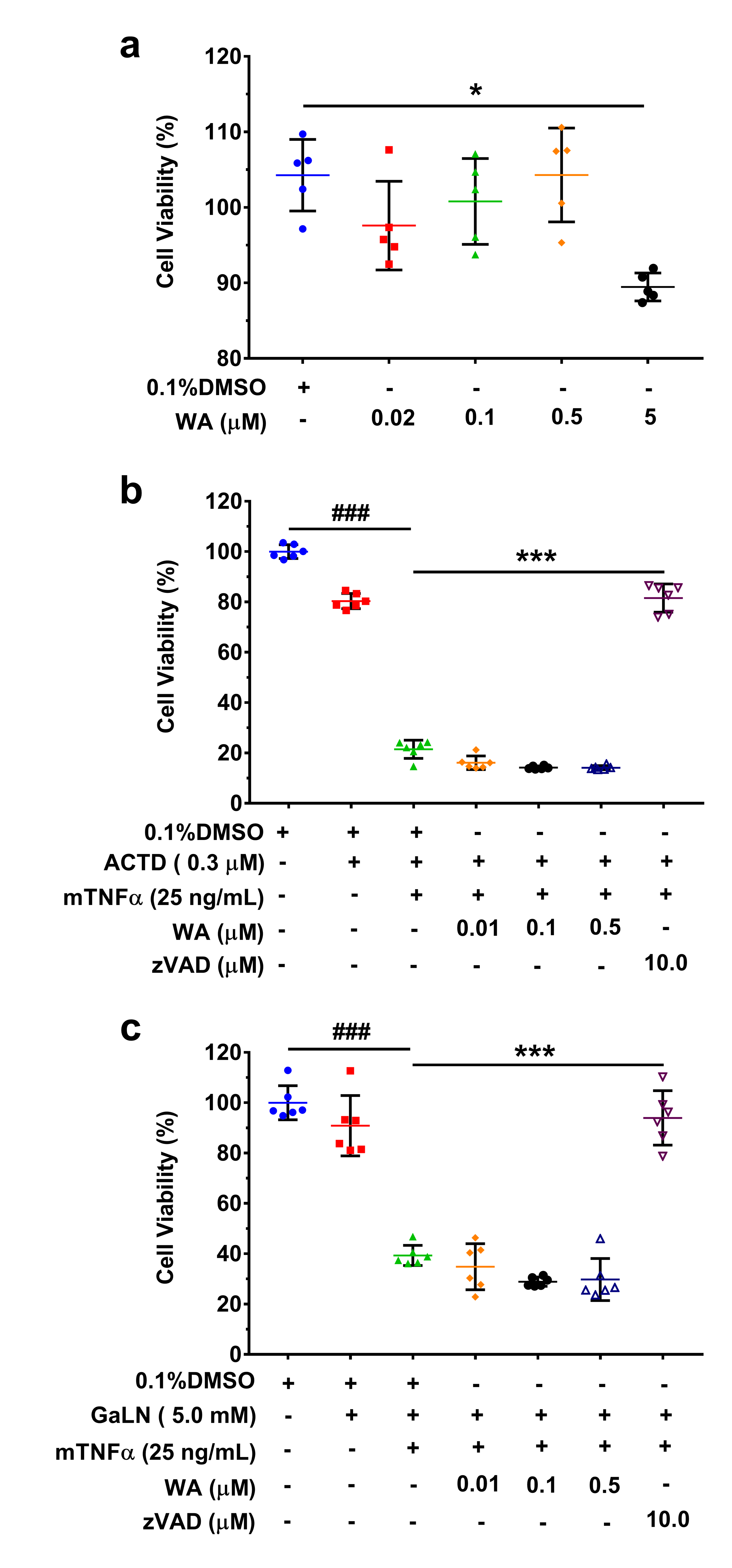

Supplement: Supplementary file 3 — Supplementary Fig. S1 [file 41419_2020_3243_MOESM3_ESM.tif]

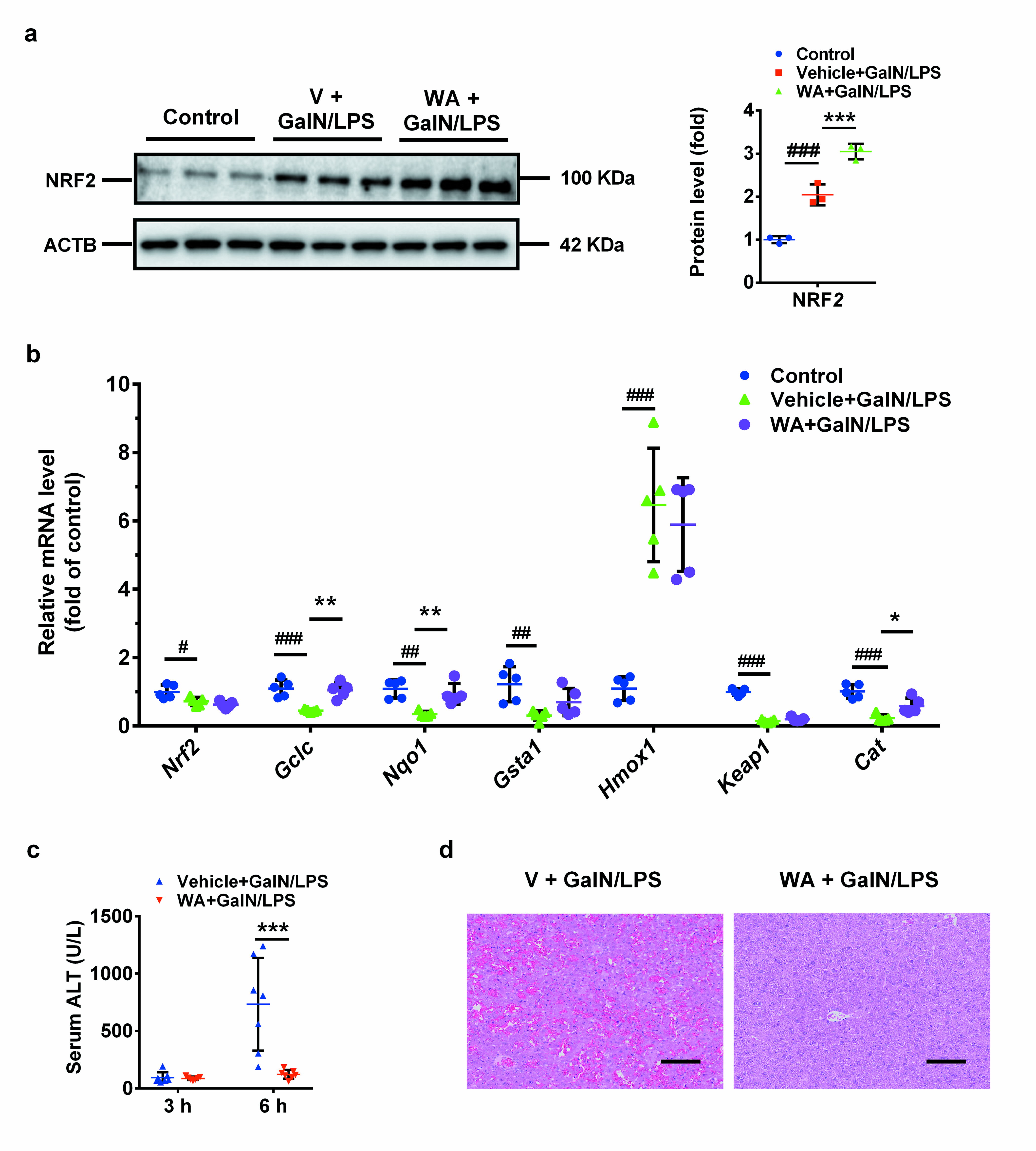

Supplement: Supplementary file 4 — Supplementary Fig. S2 [file 41419_2020_3243_MOESM4_ESM.tif]

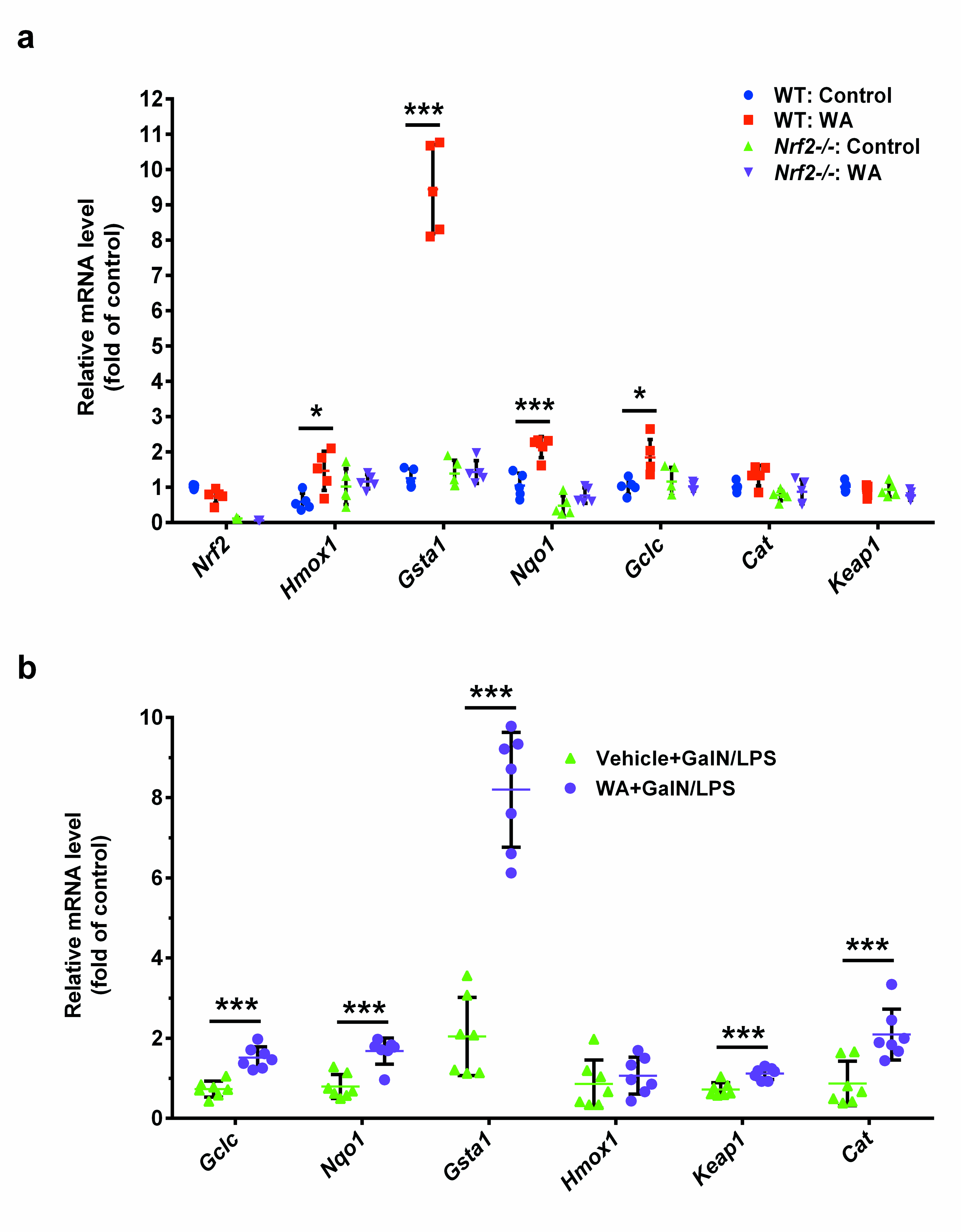

Supplement: Supplementary file 5 — Supplementary Fig. S3 [file 41419_2020_3243_MOESM5_ESM.tif]

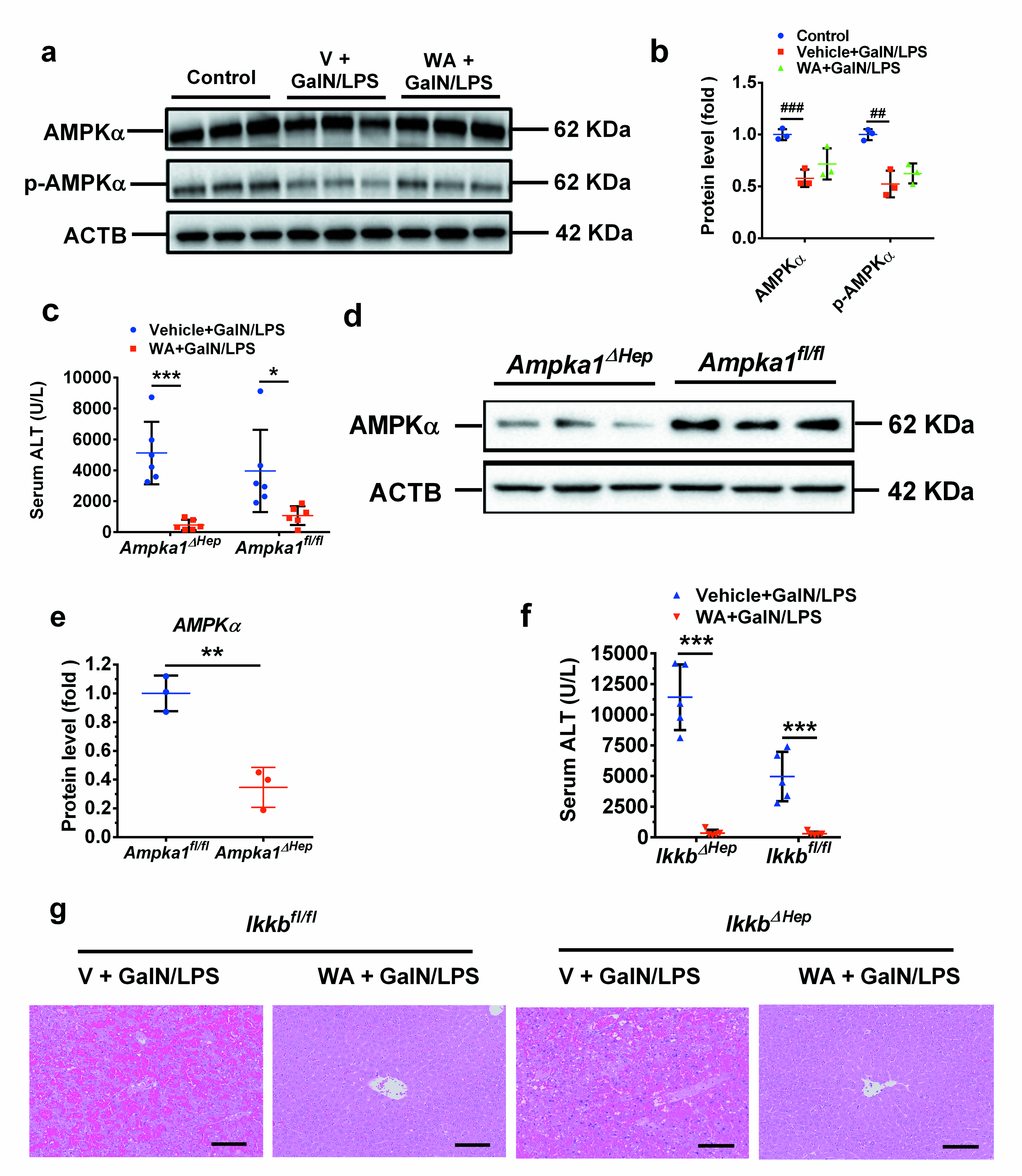

Supplement: Supplementary file 6 — Supplementary Fig. S4 [file 41419_2020_3243_MOESM6_ESM.tif]

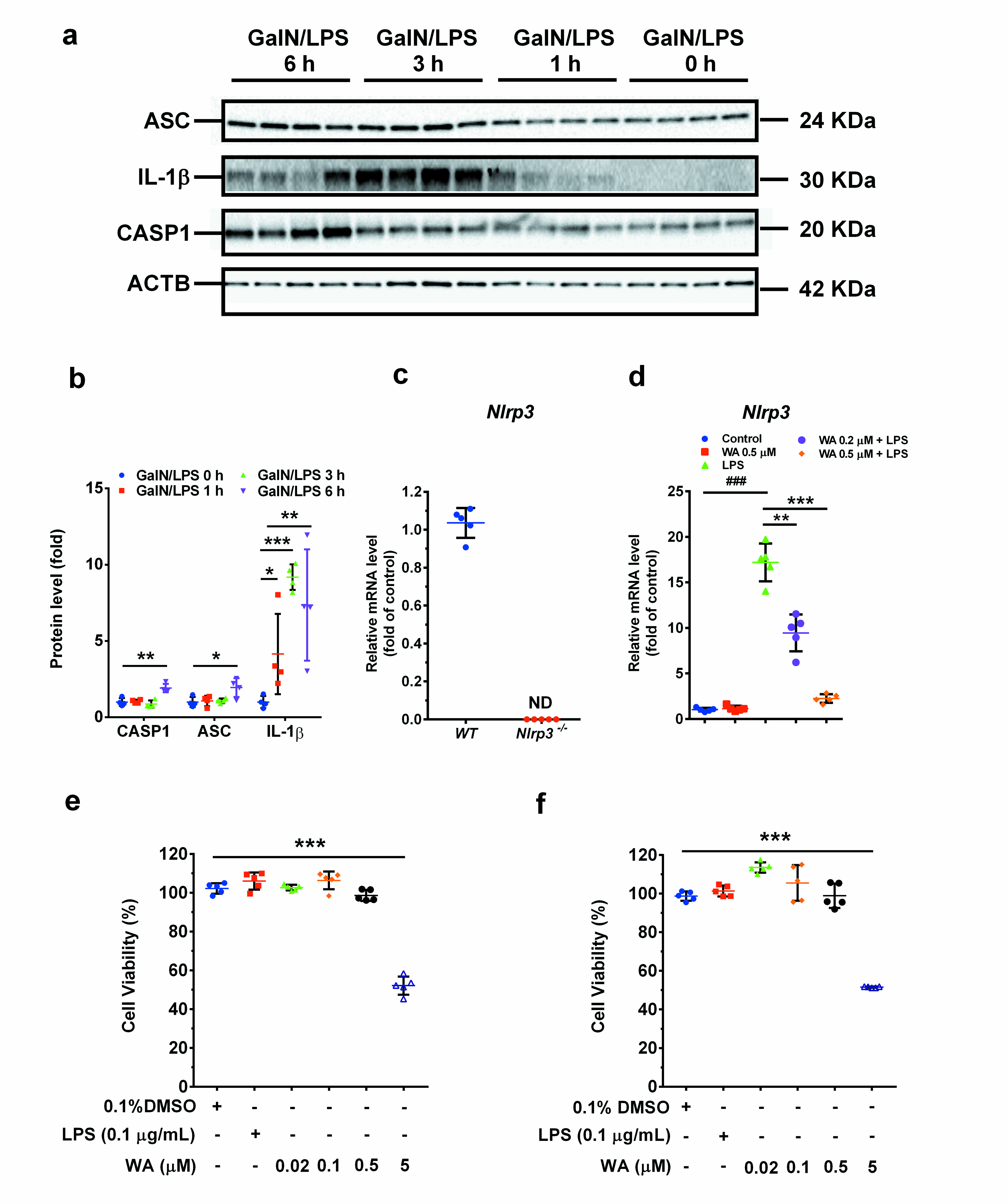

Supplement: Supplementary file 7 — Supplementary Fig. S5 [file 41419_2020_3243_MOESM7_ESM.tif]
