# Supplementary material for: Withaferin A alleviates fulminant hepatitis by targeting macrophage and NLRP3
Source: Cell Death Dis. 2021 Feb 11;12(2):174. doi: 10.1038/s41419-020-03243-w (PMC7878893; doi:10.1038/s41419-020-03243-w)
Supplement: Supplementary file 8 — Supplementary Table 1 [file 41419_2020_3243_MOESM8_ESM.pdf]

**Supplementary Table 1. Antibody Information List**

| Protein name/Antigen | Company | Catalog # | RRID |
|---|---|---|---|
| Cleaved PARP-1 | cell signaling | 9548S | AB_2160592 |
| CASP3 | cell signaling | 9664S | AB_2070042 |
| ATG3 | cell signaling | 3415S | AB_2059244 |
| LC3I/II | cell signaling | 12741S | AB_2617131 |
| AMPKα | cell signaling | 5831T | AB_10622186 |
| p-AMPKα | cell signaling | 2535T | AB_331250 |
| ASC | cell signaling | 67824S | AB_2799736 |
| pro-IL-1 $\beta$ | cell signaling | 12507S | AB_2721117 |
| CASP1 | Santa Cruz | SC56036 | AB_781816 |
| NRF2 | cell signaling | 12721T | AB_2715528 |
