# Supplementary material for: Withaferin A alleviates fulminant hepatitis by targeting macrophage and NLRP3
Source: Cell Death Dis. 2021 Feb 11;12(2):174. doi: 10.1038/s41419-020-03243-w (PMC7878893; doi:10.1038/s41419-020-03243-w)
Supplement: Supplementary file 9 — Supplementary Table 2 [file 41419_2020_3243_MOESM9_ESM.pdf]

Supplementary Table 2. List of mouse qPCR mRNA primers

Gene	Forward primer sequence (5´~3´)	Reverse primer sequence (5´~3´)
Actb	AAGTGCTTCTAGGCGGACTGTT	TTTTCTGCGCAAGTTAGGTTTTG
Il6	ACCAGAGGAAATTTTCAATAGGC	TGATGCACTTGCAGAAAACA
Il1b	GGTCAAAGGTTTGGAAGCAG	TGTGAAATGCCACCTTTTGA
Tnfa	AGGGTCTGGGCCATAGAACT	CCACCACGCTCTTCTGTCTAC
Ccl2	TTAAAAACCTGGATCGGAACCAA	GCATTAGCTTCAGATTTACGGGT
Nlrp3	TCAGATTGCTGTGTGTGGGACTGA	AGCTCAGAACCAATGCGAGATCCT
Asc	GCTGGTCCACAAAGTGTCCT	GAGCAGCTGCAAACGACTAA
Casp1	TCAGCTCCATCAGCTGAAAC	TGGAAATGTGCCATCTTCTTT
Hmox1	AAGCCGAGAATGCTGAGTTCA	GCCGTGTAGATATGGTACAAGGA
Gclc	TCAAGTGGGGTGACGAGGTG	GGTCGGATGGTTGGGGTTTG
Cat	TCCGCTCTCTGTCAAAGTGTG	AGCGACCAGATGAAGCAGTG
Gsta1	CCCCTTTCCCTCTGCTGAAG	TGCAGCTTCACTGAATCTTGAAAG
Nqo1	CATCACAGGTGAGCTGAAGGA	ACAATATCTGGGCTCAGGCG
Keap1	GCGTGGAGAGATATGAGCCA	CATACAGCAAGCGGTTGAGC
Nrf2	GGTTGCCCACATTCCCAAAC	GCAAGCGACTCATGGTCATC
F4/80	TGA CTC ACC TTG TGG TCC TAA	CTT CCC AGA ATC CAG TCT TTC C
